# Metabolic Fingerprint in Hereditary Spherocytosis Correlates With Red Blood Cell Characteristics and Clinical Severity

**DOI:** 10.1097/HS9.0000000000000591

**Published:** 2021-06-12

**Authors:** Birgit van Dooijeweert, Melissa H. Broeks, Nanda M. Verhoeven-Duif, Wouter W. van Solinge, Eduard J. van Beers, Minke A. E. Rab, Edward E. S. Nieuwenhuis, Judith J. M. Jans, Marije Bartels, Richard van Wijk

**Affiliations:** 1 Central Diagnostic Laboratory-Research, University Medical Center Utrecht, The Netherlands; 2 Department of Pediatric Hematology, University Medical Center Utrecht, The Netherlands; 3 Section Metabolic Diagnostics, Department of Genetics, University Medical Center Utrecht, The Netherlands; 4 Van Creveldkliniek, University Medical Center Utrecht, The Netherlands

Hereditary spherocytosis (HS) is the most common cause of hereditary chronic hemolytic anemia in people of northern European descent (1:2000–3000).^1^ Characterized by impaired red blood cell (RBC) membrane integrity due to disruption of the (vertical) association between the cytoskeleton and the plasma membrane, RBCs from HS patients show enhanced membrane loss and thereby surface area. As a result RBCs become spheroidal with decreased deformability, leading to premature splenic sequestration.^2^ HS is highly heterogeneous both molecularly and phenotypically. Mutations have been identified in many of the major proteins of the cytoskeleton or RBC membrane. Clinically, a broad phenotypic spectrum is recognized, ranging from asymptomatic or well-compensated anemia to severe forms requiring regular blood transfusions and splenectomy. Moreover, genotype-phenotype correlations are incompletely understood.

While RBC membrane proteins have been implicated in cellular metabolism, little is known about the metabolic consequences of defective membrane-cytoskeleton integrity in HS.^3^ Here we investigated the metabolic impact of the membrane defect in HS using untargeted metabolomics in dried blood spots (DBS). Materials and Methods are described in Section S1 (http://links.lww.com/HS/A158). We report a metabolic fingerprint for HS that provides promising leads for understanding clinical heterogeneity in patients as well as leads for further study into pathophysiological mechanisms associated with decreased membrane and cytoskeleton integrity.

In our study, we compared DBS of HS patients (n = 35) with healthy controls (HCs, n = 50). Clinical and laboratory characteristics and baseline comparison are summarized in Supplemental Table 1 (http://links.lww.com/HS/A158). The dataset comprised Z scores for 1770 unique metabolite features corresponding to 3565 metabolite annotations. The variation in metabolic fingerprints between both groups was analyzed using principal component analysis (PCA) and partial least squares discriminant analysis (PLS-DA), in which the number of features is reduced by combining them into fewer explanatory variables. PLS-DA takes group label into account (HS or HC) to maximize the variance between groups whereas PCA does not.

The PCA revealed evident separation between patients and controls. As expected, the separation was more pronounced upon PLS-DA. Both PCA and PLS-DA indicated a distinct metabolic profile for the investigated groups, with close clustering for controls and a higher level of heterogeneity in HS patients (Figure 1A and B). Metabolites contributing most to the separation of groups in PLS-DA are reflected by high Variable Importance in Projection scores. These metabolites include multiple polyamines (spermidine, spermine, N1-acetylspermidine, putrescine), (acyl)carnitines (propionylcarnitine, oleoylcarnitine, stearoylcarnitine, L-acetylcarnitine, L-palmitoylcarnitine, linoelaidylcarnitine, L-carnitine) and the glycolytic intermediates 2,3-diphosphoglycerid acid (2,3-DPG) and glyceraldehyde 3-phosphate (GA3P) (Figure 1C; Supplemental Table 2, http://links.lww.com/HS/A158). Furthermore, t test analysis identified corresponding metabolites as significantly different between patients and controls (Figure 1D; Supplemental Table 2, http://links.lww.com/HS/A158).

**Figure 1.**
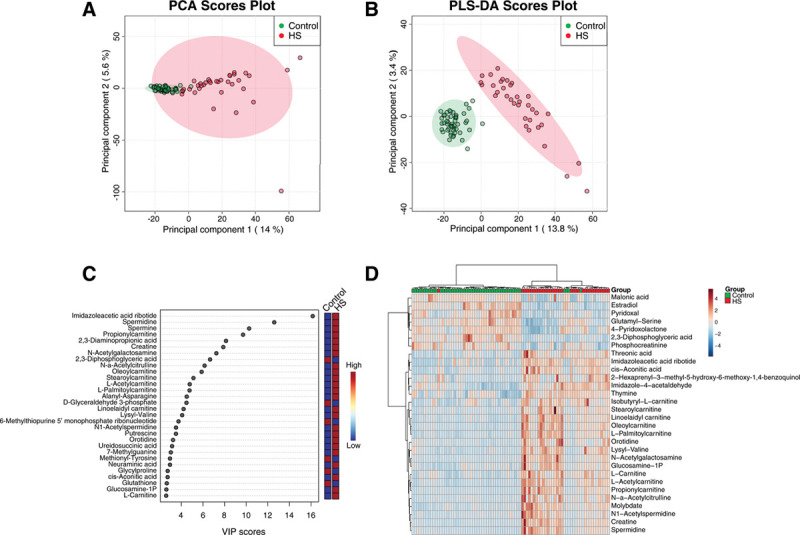
**Metabolic profile in DBS of HS patients.** (A), PCA plot and (B) PLS-DA plot of HS and HCs displayed with 95% confidence regions. Both analyses reduce dimensionality to identify the overall variation by combining all metabolite features (weighted) into new variables. These new variables are principal components. The first 2 principal components capture most of the variation in the dataset, expressed as percentage of total variation within a dataset, and are displayed on the x- and y-axis. Each dot represents a patient or control sample, colored according to group label. The position of these dots is based on the metabolite profile of 1770 unique Z scores. Samples that have a similar metabolic profile cluster more closely. The difference between PCA and PLS-DA is that PLS-DA takes group label into account to find the optimal separation between groups. (C), The metabolites that contribute the most to separation in PLS-DA are identified by a high VIP score. The 30 features that contribute the most to separation of patients and HC are displayed. (D), Heatmap of 30 most significant features identified by t test (*P* < 0.000003). Each colored cell in the heatmap corresponds to the autoscaled Z score per metabolite feature (rows). The columns of the heatmap are colored by group label. The heatmap was created with hierarchical Ward’s linkage clustering using Euclidean distances. The dendrogram represents clustering of samples and metabolite features. Figures were created using MetaboAnalyst. A comprehensive overview of *P* values and isomers is displayed in Supplemental Table 2 (http://links.lww.com/HS/A158). DBS = dried blood spot; HC = healthy control; HS = hereditary spherocytosis; PCA = principal component analysis; PLS-DA = partial least squares discriminant analysis; VIP = Variable Importance in Projection.

Based on the observation of significantly decreased 2,3-DPG and GA3P in both PLS-DA and t test, the glycolytic pathway was explored in detail. Apart from a decrease in 2,3-DPG and GA3P, none of the glycolytic intermediates were significantly altered, and no substantial differences were observed in pyruvate and lactate, or glucose 6-phosphate as respective end- and starting points of glycolysis (Supplemental Figure 1, http://links.lww.com/HS/A158). Interestingly, previous studies have shown that glycolytic enzymes associate with the RBC membrane^3,4^ and others have reported specifically on decreased pyruvate kinase (PK) activity in HS patients as a consequence of loss of membrane-bound PK.^5,6^ Here, we only found 2,3 DPG and GA3P (and/or features with the same respective mass to charge ratio; Supplemental Table 2, http://links.lww.com/HS/A158) to be significantly decreased in HS patients. This suggests that although glycolytic enzymes might be affected to some extent by decreased RBC-membrane integrity, glycolysis in general appears not strongly affected. While the metabolic profile was obtained from whole blood, it is highly likely that the observed glycolytic disturbances mainly reflect RBCs, since 2,3-DPG is specific to the RBC. In addition, no correlation was observed for 2,3-DPG and GA3P with any of the full blood count parameters.

While the mechanism underlying the decrease in 2,3-DPG in HS remains to be determined, it is intriguing, as 2,3-DPG is an important regulator of hemoglobin oxygen affinity. It binds with greater affinity to deoxygenated hemoglobin than to oxygenated hemoglobin and decreases the p50, thereby promoting the release of oxygen to tissues. While the reported increase of 2,3 DPG in hereditary anemias like pyruvate kinase deficiency (PKD)^7^ and beta-thalassemia minor^8^ is assumed to be clinically beneficial for patients, the robust decrease that we observe in HS patients could also be of clinical importance. In this respect, the reported fatigue-related impairment of quality of life, and “complaints of fatigue” being reported as critically factored into the decision for splenectomy in a substantial number of HS patients despite relatively mild to moderate hemolysis, might be well worth further exploring.^9,10^

Further exploration of red cell metabolism revealed decreased glutathione, but no other metabolites of glutathione metabolism were altered (data not shown). In addition, the pentose phosphate pathway and its respective metabolites appeared unaffected (data not shown). Furthermore, investigation of amino acids and carnitines revealed a significant decrease in aspartate, and significant increases in glycine, L-valine, L-carnitine and L-acetylcarnitine (data not shown). Putrescine, spermidine and spermine, players of arginine and polyamine metabolism, were significantly elevated in HS patients compared to controls (Supplemental Figure 2, http://links.lww.com/HS/A158).

To explore whether the heterogeneity in HS metabolic profiles related to clinical phenotype, additional PCA and PLS-DA were performed. Clinical phenotypes were classified as mild, moderate or severe, based on the severity of anemia and degree of compensation for hemolysis, according to the modified criteria originally proposed by Eber et al.^11^ The PLS-DA plot showed that mildly affected HS patients clustered most closely to controls, followed by moderately affected patients, whereas the metabolic profile from severely affected individuals differed most from controls (Figure 2A and B). As the clinical phenotype in HS is highly heterogeneous and severity categories depend upon 4 parameters only (hemoglobin, reticulocyte count, bilirubin and splenectomy), the distinction is arbitrary and subject to fluctuation over time. This is likely reflected by the heterogeneity in the “moderately affected” HS-group (Supplementary Table 1, http://links.lww.com/HS/A158). Nevertheless, a clear distinction is observed between mildly or even moderately affected HS patients and severely affected HS patients.

**Figure 2.**
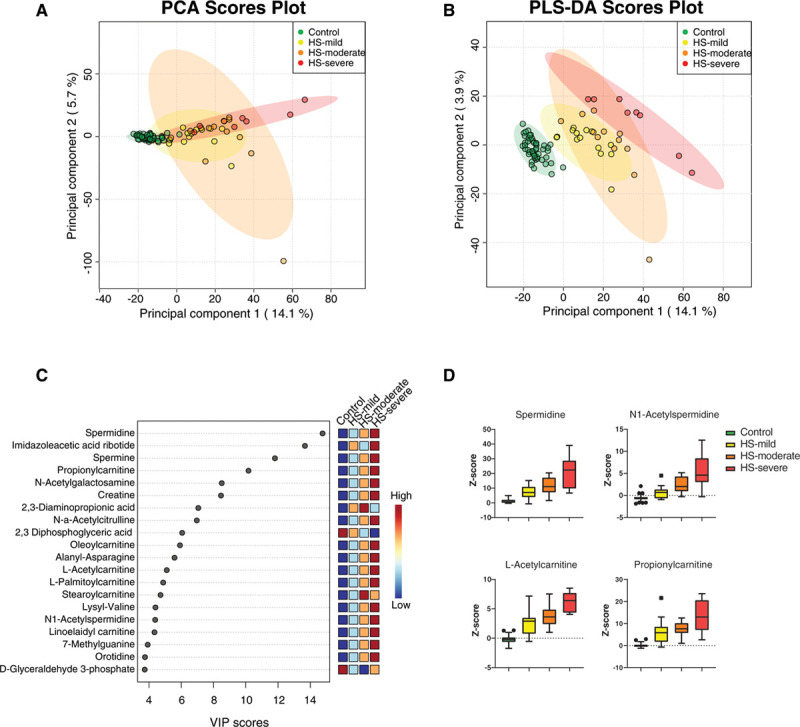
**Metabolic profile in relation to clinical severity phenotypes.** (A), PCA plot and (B) PLS-DA plot distinguishing clinical severity. (C), Top 20 features contributing to the separation of patients and controls in PLS-DA, reflected by VIP-scores. For almost all metabolites a correlation with clinical severity is observed, reflected by an increasing or decreasing color gradient. (D), Z scores of spermidine, N1-Acetylspermidine, L-Acetylcarnitine and Propionylcarnitine based on clinical severity for control (n = 50), HS-mild (n = 15), HS-moderate (n = 12) and HS-severe (n = 8) in a boxplot with Tukey whiskers. HS = hereditary spherocytosis; PCA = principal component analysis; PLS-DA = partial least squares discriminant analysis; VIP = Variable Importance in Projection.

Metabolites that contributed the most to the distinction between HC and the clinical severity of HS included polyamines, (acyl)carnitines, 2,3-DPG and creatine (Figure 2C). Increased clinical severity was associated with increased Z scores of spermidine, N1-Acetylspermidine, L-Acetylcarnitine and propionylcarnitine (Figure 2D). In addition, analysis of these metabolites in relation to full blood count parameters revealed significant correlations for spermidine, N1-acetylspermidine, L-acetylcarnitine and propionylcarnitine with RBC and reticulocyte counts (Supplemental Figure 3, http://links.lww.com/HS/A158). Together with the distinct profiles for clinical severity subgroups, the gradient that was demonstrated for top metabolites indicates that clinical phenotypes correlate with the metabolic profiles of HS-patients. While these findings need to be validated in a larger cohort of patients, our approach offers new perspectives for a better understanding of the complex genotype-phenotype associations in HS.

Lastly, we explored the correlation of the top 20 features of PLS-DA with functional features of RBC deformability as established with osmotic gradient ektacytometry. For the maximum deformability of RBCs, reflected by the maximum elongation index (EI max), an inverse correlation with Z scores of spermidine (*r* = –0.37; *P* = 0.04), N1-Acetylspermidine (*r* = –0.47; *P* = 0.008), L-Acetylcarnitine (*r* = –0.42; *P* = 0.02), and propionylcarnitine (*r* = –0.36; *P* = 0.05) (Supplemental Figure 3, http://links.lww.com/HS/A158) was observed. Further correlations with functional features of RBC deformability were observed between propionylcarnitine and the total area under the curve (*r* = –0.37; *P* = 0.04) and between L-carnitine and O hyper (*r* = 0.37; *P* = 0.04, plots not shown), suggesting that increases in distinctive metabolites were associated with decreased RBC hydration and deformability. These findings were supported by the relation between the top metabolites and change in deformability (ΔEI) as measured with the cell membrane stability test in a subset of HS patients (n = 8), where we observed a strong correlation for propionylcarnitine and ΔEI (*r* = 0.74; *P* = 0.05).

Previously, the polyamines spermidine, spermine and putrescine have been reported to decrease erythrocyte membrane deformability and stabilize the membrane skeleton of resealed ghosts loaded with polyamines.^12^ The inverse correlation between spermidine and N1-Acetylspermidine and the maximal deformability (EI max) of RBCs in HS patients, although not very strong, as well as the relation with clinical phenotypes we observe in this study, provides supporting evidence for the hypothesis that apart from an in vitro-effect in ghosts, the concentration of polyamines is associated with in vivo red cell deformability in patients. Whether this contributes to the decreased deformability seen in HS, or is a compensatory mechanism, remains to be determined.

The observed correlations between polyamines and reticulocyte count, and inverse correlations with hemoglobin and erythrocyte count, but not with leukocytes and platelets, suggests that these alterations are RBC-specific. Interestingly, previous studies demonstrated significant correlations between younger and older RBCs, with lower levels of polyamines in the older red cells, suggesting the possibility of using red cell polyamines as an indicator of the activity of the bone marrow in anemic states.^13^ The increase of polyamines we observed previously in PKD,^14^ another hemolytic anemia with a hyper-regenerative bone marrow, further supports these findings. As concentrations of polyamines have also been reported to be increased in RBC’s and DBS in sickle cell disease and PKD,^14,15^ we anticipate that unraveling the underlying mechanisms and consequences of altered polyamine metabolism may contribute not only to a better understanding of HS pathophysiology but for rare hereditary hemolytic anemia in general.

In summary, we report on a metabolic fingerprint that offers for the first time a comprehensive overview of metabolic disturbances in HS and includes altered levels of polyamines and (acyl)carnitines. These metabolic disturbances correlate with RBC characteristics (in particular deformability) and, in addition, to clinical severity. We also identified significant decreases in glycolytic intermediates 2,3-diphosphoglyceric acid and glyceraldehyde-3-phosphate. We demonstrate that untargeted metabolomics can be instrumental in investigating the phenotypic heterogeneity in patients and our results provide promising leads for further study into the pathophysiological mechanisms that determine the phenotypic expression of HS. Lastly, this comprehensive characterization of metabolic disturbances in HS, might serve as a starting point for the development of new therapeutic strategies.

## Acknowledgments

We thank Nienke van Unen and Fini de Gruyter for their technical support in Bio-informatics.

## Disclosures

EJvB and RvW perform consultancy and receive research funding from Agios Pharmaceuticals. All the other authors have no conflicts of interest to disclose.

## Sources of funding

This study was supported in part by research funding from MetaKids (Grant No. 2017-075) to JJMJ.

## Supplementary Material


