# Supplementary material for: Metabolic Fingerprint in Hereditary Spherocytosis Correlates With Red Blood Cell Characteristics and Clinical Severity
Source: Hemasphere. 2021 Jun 12;5(7):e591. doi: 10.1097/HS9.0000000000000591 (PMC8196084; doi:10.1097/HS9.0000000000000591)
Supplement: Supplementary file 1 [file hs9-5-e591-s001.docx]

**Supplemental Figures and Tables:**

| **Supplemental Table S1A. Clinical characteristics of HS patients and baseline comparison to healthy controls** | | | | | | | | | | | | | | | |
| --- | --- | --- | --- | --- | --- | --- | --- | --- | --- | --- | --- | --- | --- | --- | --- |
| **Age (years)** | **Gender** | **Hb (mmol/L)** | **RBC (x10^12^/L)** | **Retics (x10^9^/L)** | **WBC (x10^9^/L)** | **Plts (x10^9^/L)** | **Treatment** | **Allele 1** | | **Allele 2** | | **Severity category** | |  |  |
| 8 | female | 5.7 | 3.01 | 485 | 8.9 | 225 | splenic embolization (50% residu) | ND | | ND | | Severe | |  |  |
| 8 | male | 5.6 | 3.21 | 434 | 5.1 | 268 | sporadic transfusions | SPTA1: c.4339-99C>T; p.(?) | | SPTA1: c.6769G>T; p.(Glu2257*) | | Severe | |  |  |
| 1.5 | male | 3.9 | 2.50 | 484 | 15.7 | 280 | no current treatment | SPTA1: c.1850dup; p.(Ser618fs) | | SPTA1: c.4339-99C>T; p.(?) | | Severe | |  |  |
| 0.2 | female | 4.2 | 2.33 | 350 | 14.7 | 742 | sporadic transfusions/no current | ANK1: c.2390_2393del; p.(Leu797fs) | | SPTA1: c.6889; p.(Arg2297Trp) | | Severe | |  |  |
| 12 | male | 7.3 | 4.04 | 433 | 6.5 | 340 | no current treatment | ANK1: c.462C>T; p.(Arg1488*) | | SPTA1: c.6531-12C>T; p(?) | | Severe | |  |  |
| 58 | male | 7.6 | 3.55 | 441 | 5.9 | 148 | splenectomy; no current treatment | SLC4A1: c.1225G>T; p.(Val409Phe) | | x | | Severe | |  |  |
| 43 | female | 7.1 | 3.45 | 350 | 4.6 | 169 | splenectomy; no current treatment | SLC4A1: c.2494C>T; p.(Arg832Cys) | | x | | Severe | |  |  |
| 14 | male | 8.6 | 4.58 | 624 | 6.1 | 274 | no current treatment | ND | | ND | | Severe | |  |  |
| 64 | male | 5.9 | 3.00 | 212 | 6.0 | 276 | no current treatment | SPTA1: c.4339-99C>T; p.(? | | SPTA1: c.6989G>A; p.(Arg2330Lys) | | Moderate | |  |  |
| 4 | male | 6.4 | 3.55 | 348 | 21.1 | 232 | sporadic transfusions | ND | | ND | | Moderate | |  |  |
| 0.7 | male | 5.8 | 3.53 | 341 | 9.6 | 331 | no current treatment | ND | | ND | | Moderate | |  |  |
| 2 | male | 7.5 | 3.87 | 386 | 11.4 | 367 | no current treatment | ND | | ND | | Moderate | |  |  |
| 39 | female | 9.0 | 4.94 | 313 | 8.4 | 365 | splenectomy; no current treatment | SPTB: c.4978C>T; p.(Gln1660*) | | x | | Moderate | |  |  |
| 6 | female | 7.2 | 3.86 | 369 | 7.7 | 488 | no current treatment | ANK1: c.3123del; p.(Ser1042fs) | | x | | Moderate | |  |  |
| 30 | female | 5.7 | 2.83 | 201 | 3.6 | 138 | splenectomy; no current treatment | SPTA1: c.3527dup; p.(Leu1086fs) | | SPTA1: c.6531-12C>T; p(?) | | Moderate | |  |  |
| 4 | female | 6.8 | 3.64 | 389 | 10.7 | 301 | no current treatment | SPTB: c.1714_1723del; p.(Met573fs) | | x | | Moderate | |  |  |
| 0.3 | male | 5.0 | 3.21 | 298 | 12.9 | 505 | sporadic transfusions | ND | | ND | | Moderate | |  |  |
| 3 | male | 5.4 | 3.02 | 553 | 8.0 | 376 | regular transfusions | SPTB: c.2889C>A; p.(Cys963*) | | x | | Moderate | |  |  |
| 2 | male | 7.1 | 4.53 | 150 | 10.5 | 428 | no current treatment | SPTA1: c.134G>C; p.(Arg45Thr) | | SPTA1: c.6531-12C>T; p(?) | | Mild | |  |  |
| 73 | male | 7.4 | 3.49 | 143 | 4.6 | 158 | no current treatment | SPTA1: c.5805G>A; p.(Tryp1935*) | | SPTA1: c.6531-12C>T; p(?) | | Mild | |  |  |
| 0.8 | male | 6.9 | 4.15 | 158 | 10.5 | 558 | no current treatment | ANK1: c.4391-2@>G; p.(?) | | SPTA1: c.4339-99C>T; p.(?) | | Mild | |  |  |
| 9 | male | 8.8 | 5.49 | 162 | 8.7 | 615 | splenic embolization/coiling | ANK1 c.2563_2586delinsCCAG p.(Glu855fs) | | x | | Mild | |  |  |
| 4 | male | 7.5 | 4.24 | 298 | 9.6 | 360 | no current treatment | SPTB: c.4978C>T; p.(Gln1660*) | | x | | Mild | |  |  |
| 27 | male | 9.4 | 5.42 | 137 | 9.4 | 307 | splenectomy; no current treatment | ANK1: c.23dup; p.(Glu9fs) | | x | | Mild | |  |  |
| 9 | male | 8.1 | 4.56 | 188 | 7.6 | 314 | no current treatment | ANK1: c.4559del; p.(Glu1520fs*) | | x | | Mild | |  |  |
| 7 | male | 7.5 | 4.15 | 128 | 6.9 | 290 | no current treatment | SPTA1: c.6788+1G>A; p.(?) | | SPTA1: c.6531-12C>T; p(?) | | Mild | |  |  |
| 6 | male | 7.5 | 4.32 | 560 | 8.1 | 436 | sporadic transfusions | SPTB: c.5128G>T; p.(Glu1710*) | | x | | Mild | |  |  |
| 13 | male | 8.7 | 4.94 | 87.4 | 17.1 | 620 | splenectomy; no current treatment | SPTB: c.5898C>T; p.(=) | | x | | Mild | |  |  |
| 65 | female | 8.0 | 4.06 | 275 | 5.6 | 252 | no current treatment | SPTA1: c.6788+1G>A ; p.(?) | | SPTA1: c.6531-12C>T; p(?) | | Mild | |  |  |
| 5 | female | 7.6 | 5.70 | 162 | 14.1 | 812 | splenectomy; no current treatment | SPTA1: c.83G>A; p.(Arg28His) | | SPTA1: c.6531-12C>T; p(?) | | Mild | |  |  |
| 7 | male | 8.0 | 6.03 | 124 | 14.8 | 911 | splenectomy; no current treatment | SPTA1: c.83G>A; p.(Arg28His) | | SPTA1: c.6531-12C>T; p(?) | | Mild | |  |  |
| 21 | female | 8.1 | 4.02 | 444 | 6.8 | 244 | no current treatment | SLC4A1: c.616_620delG | | x | | Mild | |  |  |
| 9 | male | 8.0 | 4.33 | 245 | 4.0 | 181 | no current treatment | SLC4A1: c.2608C>T; p.(Arg870Trp) | | x | | Mild | |  |  |
| 2 | male | 7.1 | 4.16 | 245 | 9.0 | 443 | no current treatment | SPTB: c.4117C>T; p.(Gln1373*) | | x | | Mild | |  |  |
| 70 | female | ND | ND | ND | ND | ND | no current treatment | ANK1: c.4638_4639del; p.(Leu1547fs) | | x | | ND | |  |  |
| **Normal:** | | 7.4-10.7 | 3.6-5.5 | 25-120 | 4.0-13.5 | 150-450 | ; * Age and gender dependent | |  | |  | |  | |  |

| **Table S1B. Baseline comparison to controls** | | | | |  |  | |
| --- | --- | --- | --- | --- | --- | --- | --- |
|  |  |  | **HS (mean ±SD)** | **HC (mean ±SD)** | | |  |
| Age (years) | | | 17.9 ± 22.5 | 38.4 ± 11.7 | | |  |
| Hb (mmol/L) | | | 7.07 ± 1.33 | 9.07 ± 0.75 | | |  |
| Retics (x10^9^/L) | | | 309.3 ± 143.7 | 58.1 ± 17.7 | | |  |
| Time to DBS (hours) | | | 3.92 ± 1.9 | 3.97 ± 6.7 | | |  |

**Supplemental Table S1. Clinical characteristics of HS patients and baseline comparison to healthy controls.
A.** Clinical characteristics of HS patients regarding age, gender, hemoglobin (Hb), red blood cell count (RBC), reticulocyte count (Retics), white blood cell count (WBC), platelets (Plts), treatment, genetics diagnostics and severity category. Sporadic transfusions are defined as ≤ 6 per 12 months. ND = not determined. x = no additional defect was identified. **B.** Comparison of age, Hb, Retics and time between blood withdrawal and spotting (time to DBS) between healthy controls (HC) and HS patients. Data are presented as mean ± SD.

**
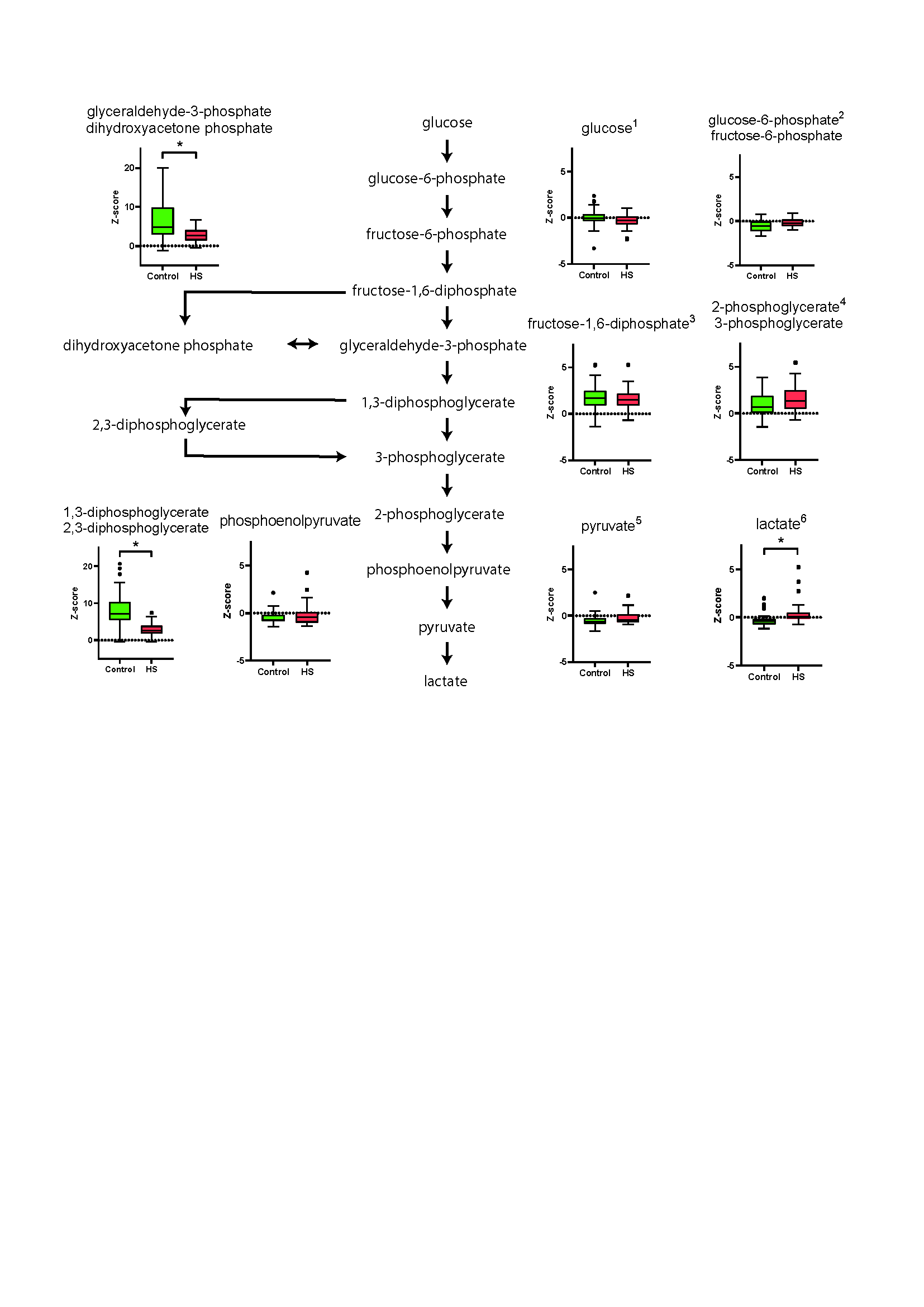
Supplemental Figure S1. Glycolytic intermediates in HS versus controls.**

**Legend of Supplemental Figure S1. Glycolytic intermediates in HS versus controls.**Schematic representation of glycolysis in red blood cells. Z-scores of glycolytic intermediates (and glycolytic isomers) are plotted for healthy control (n=50) and HS-samples (n=35) in a boxplot with Tukey whiskers. Additional isomers of glycolytic intermediates include: 1) D-Galactose, D-Mannose, Myoinositol, 3-Deoxyarabinohexonic acid, Beta-D-Glucose, D-Fructose, Allose, L-Sorbose, Alpha-D-Glucose, D-Tagatose, Beta-D-Galactose, Scyllitol, L-Gulose, L-Galactose. 2) Myo-inositol 1-phosphate, Galactose 1-phosphate, Dolichyl phosphate D-mannose, Fructose 1-phosphate, Mannose 6-phosphate, D-Myo-inositol 4-phosphate, Glucose 1-phosphate, Inositol phosphate, Beta-D-Glucose 6-phosphate, Beta-D-Fructose 6-phosphate, D-Tagatose 1-phosphate, D-Mannose 1-phosphate, Sorbose 1-phosphate, Beta-D-Fructose 2-phosphate, 1D-myo-Inositol 3-phosphate, D-Tagatose 6-phosphate, D-fructose 1-phosphate. 3) 1D-Myo-inositol 1,4-bisphosphate, D-fructose 2,6-bisphosphate, Alpha-D-Glucose 1,6-bisphosphate, 1D-Myo-inositol 1,3-bisphosphate, 1D-Myo-inositol 3,4-bisphosphate, D-Tagatose 1,6-bisphosphate, D-Mannose 1,6-bisphosphate, beta-D-Fructose 1,6-bisphosphate. 4) 2-Phospho-D-glyceric acid, (2R)-2-Hydroxy-3-(phosphonatooxy)propanoate. 5) Malonic semialdehyde.6) Hydroxypropionic acid, Glyceraldehyde, D-Lactic acid, Dihydroxyacetone, Methoxyacetic acid. * p<0.0001.

**Supplemental Figure 2. Arginine & polyamine metabolism plots.**

**
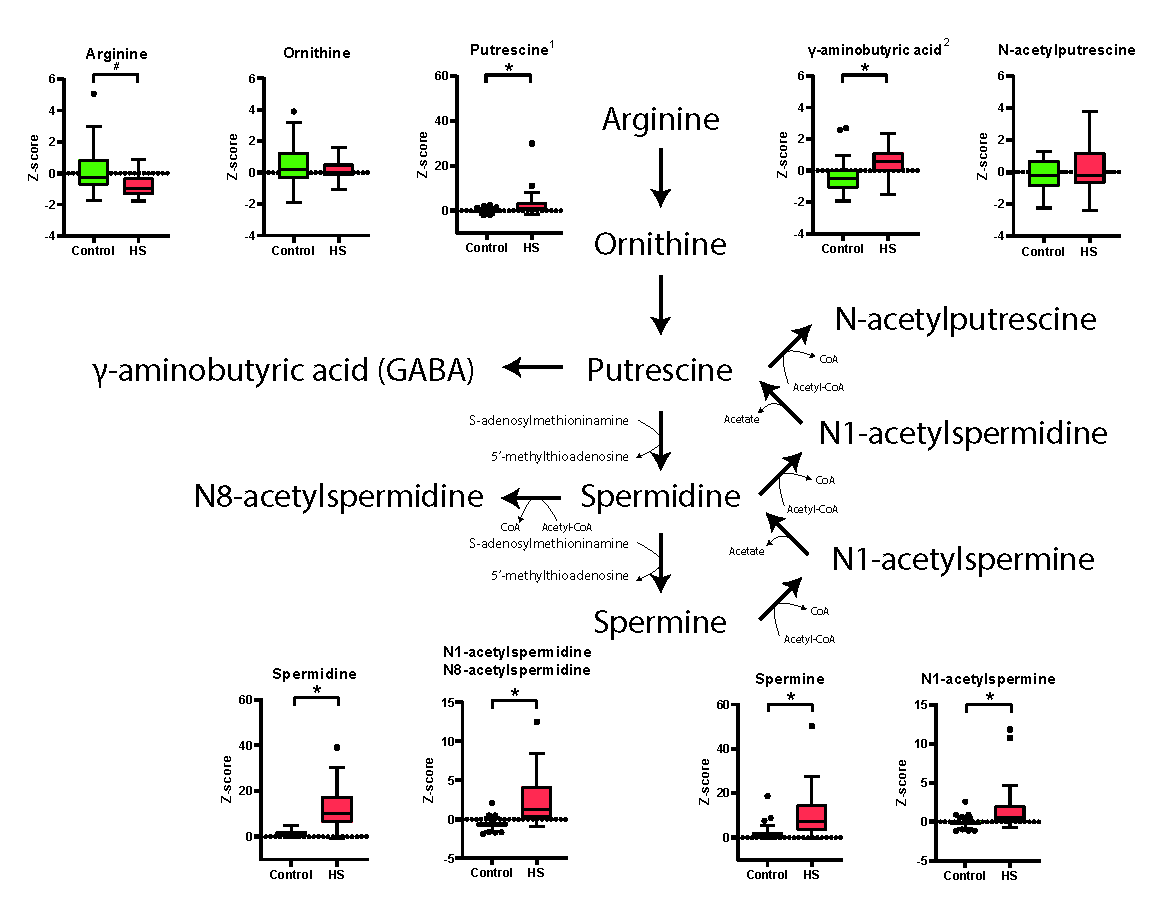
**

**Legend of Supplemental Figure S2. Arginine & polyamine metabolism plots.**Schematic view of arginine and polyamine metabolism. Z-scores for metabolites in polyamine metabolism are plotted for healthy control (n=50) and HS-samples (n=35) in a boxplot with Tukey whiskers. Arginine (p=0.0002), Ornithine (p=0.3702), Putrescine (p<0.0001), y-aminobutyric acid (p<0.0001), N-acetylputrescine (p=0.5531), Spermidine (p<0.0001), N1/N8-acetylspermidine (p<0.0001), Spermine (p<0.0001), N1-acetylspermine (p<0.0001). Isomers include 1) 1,4-Butanediammonium. 2) Dimethylglycine, L/D-Alpha-aminobutyric acid, 2-Aminoisobutyric acid, (S)-b-aminoisobutyric acid, (R)-b-aminoisobutyric acid, 3-Aminoisobutanoic acid, 3-Aminobutanoic acid, N-Ethylglycine. * p<0.0001, # p<0.001.

**
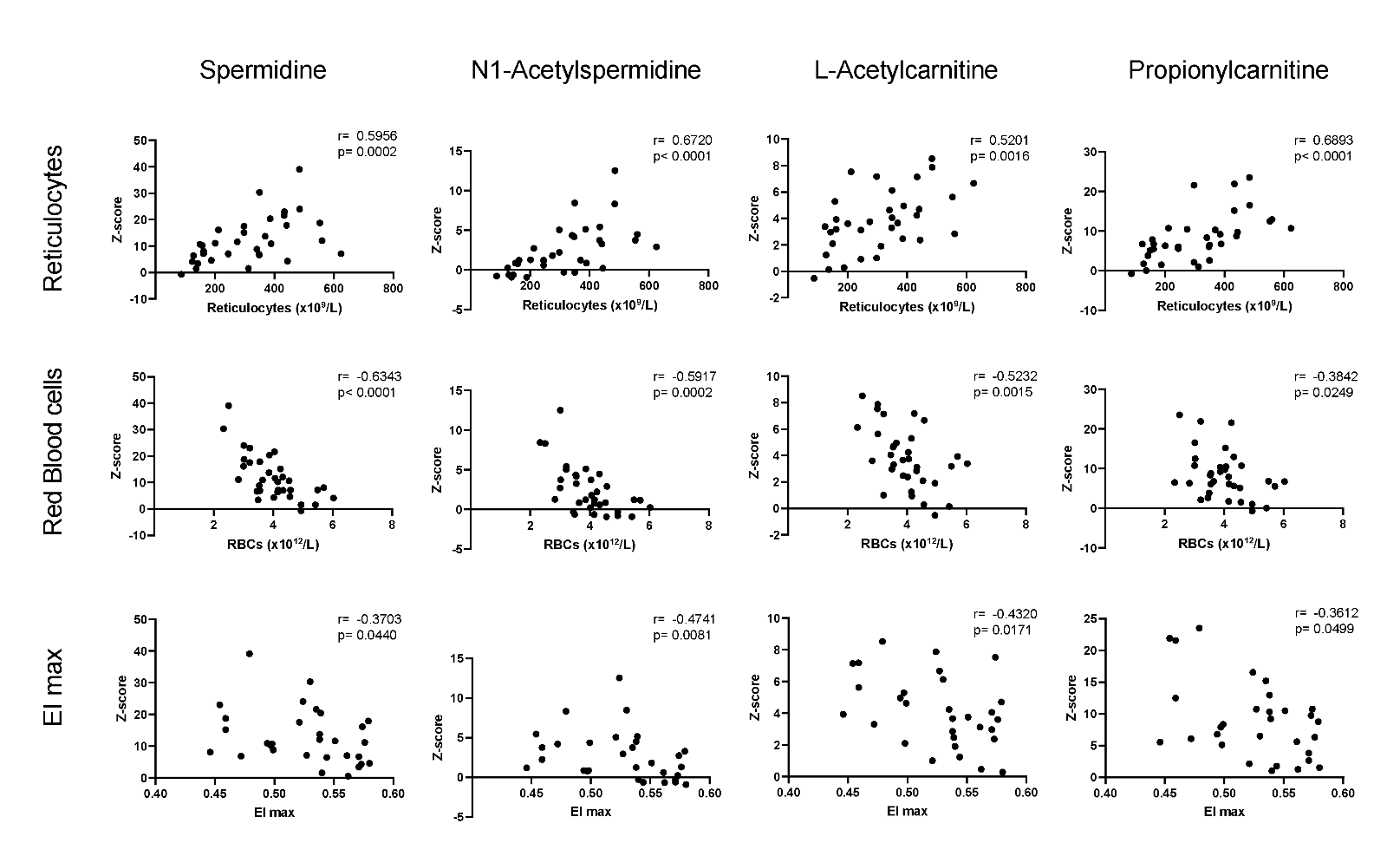
Supplemental Figure 3. Correlation of distinctive metabolites with red cell characteristics & deformability.**

**Legend of Figure S3. Correlation of distinctive metabolites with red cell characteristics & deformability.**Correlations between number of reticulocytes (n=34), red blood cells (n=34), EI max (n=30) and Z-scores of spermidine, N1-Acetylspermidine, L-Acetylcarnitine and Propionylcarnitine in HS patients.

**SDC Section S1: Materials and methods**

*Patients and samples*

Thirty-five patients diagnosed with HS were included. Diagnosis was based on full blood count, general markers of hemolysis, family history and functional testing (osmotic gradient ektacytometry, eosin-5’-maleimide binding and the osmotic fragility test). Diagnosis was confirmed in the majority of cases by next generation sequencing gene panel analysis. Healthy volunteers (institutional blood donor service) served as controls (HC). All patients or their legal guardians approved the use of left-over material for method development and validation, in agreement with institutional and national legislation. All procedures followed were in accordance with the Helsinki Declaration of 1976, as revised in 2000, and the ethical standards of the University Medical Center Utrecht (University Medical Center Utrecht Biobank Regulations; version June 19^th^ 2013). For DBS, 50 µL aliquots of whole blood (EDTA) were spotted onto Guthrie card filter paper (Whatman no. 903 Protein Saver TM cards). All spots were left to dry for at least four hours at room temperature, and were subsequently stored at -80 ⁰C in a foil bag with a desiccant package pending further analysis.

*Untargeted metabolic phenotyping*

Sample preparation, direct infusion high resolution mass spectrometry (DI-HRMS) and data processing were performed as previously reported.^(1-3)^ Mass peak intensities for metabolite annotation were averaged over technical triplicates. As DI-HRMS is unable to separate isomers, mass peak intensities consisted of summed intensities of isomers. Metabolite annotation was performed using a peak calling bioinformatics pipeline developed in R programming software, based on the human metabolome database (version 3.6 (Wishart et al.))(<http://github.com/UMCUGenetics/DIMS>).

DBS-samples were distributed over several DI-HRMS runs. To each DI-HRMS run an extra set of control samples was added (spotted from heparin blood). To compare the metabolic profiles of HS and HC between runs, mass peak intensities for each identified feature were converted to Z-scores. These scores, based on the extra control samples, were calculated by the following formula:

$$Z-score=\frac{(Mass peak intensity of Pt or HC sample-Mean mass peak intensities of extra control samples}{Standard deviation mass peak intensities of extra control samples\ddagger}$$

‡Extra controls exist of a batch of banked DBS samples from individuals in whom an inborn error of metabolism (IEM) was excluded after an extensive diagnostic workup.

*Data analysis*

Z-scores calculated from several DI-HRMS runs were combined to a final metabolomics dataset. Data analyses were conducted in MetaboAnalyst. No further data filtering or normalization was applied. Outlying metabolite features were identified using the PCA loadings plot. In total 10 outlying features were removed, resulting in a final dataset of 1770 unique features corresponding to 3565 metabolite annotations. Multivariate PCA and PLS-DA analysis were conducted, as well as a two-sample t-test with equal group variance. Additional analyses were performed in Graphpad Prism (Version 8.3.0;538). Individual Z-scores of features were compared using the Mann Whitney test. Correlations of Z-scores and blood characteristics were analyzed using Spearman Rank. Bonferroni correction for multiple testing was applied, considering p-values statistically significant when p<0.001.

*Ektacytometry*


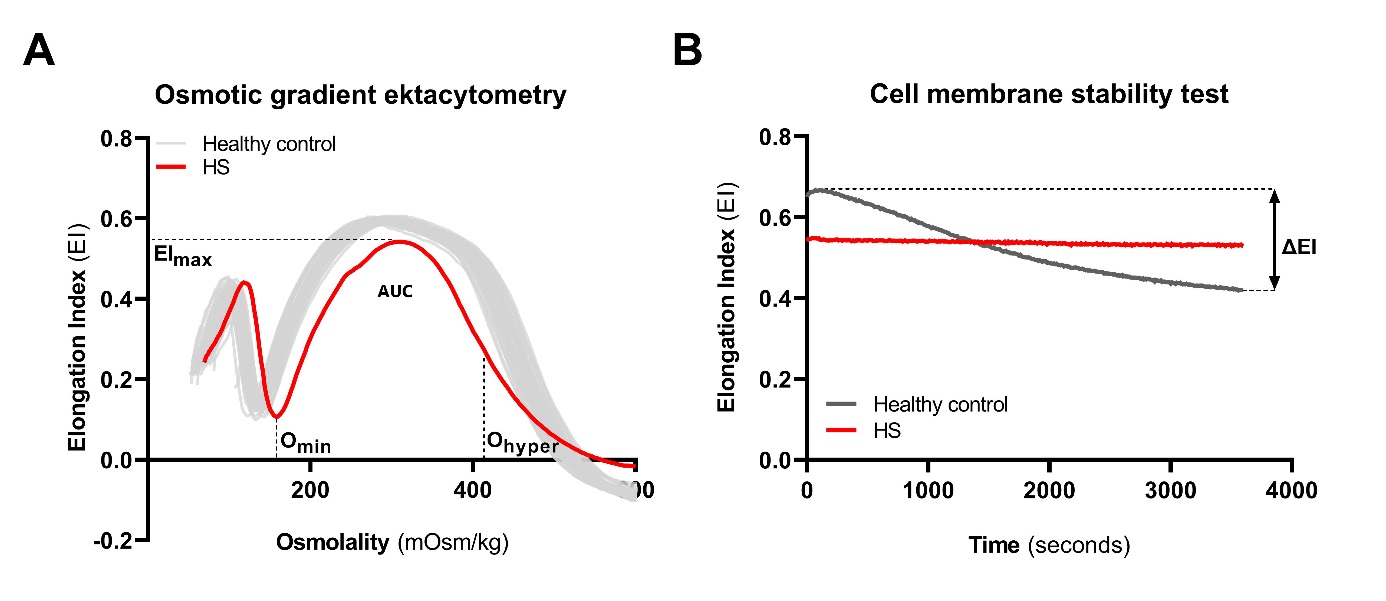
Osmotic gradient ektacytometry data were available for 30 of 35 HS patients. This functional test of RBC deformability is performed using the Laser optical rotational red cell analyzer (Lorrca, RR Mechatronics, Zwaag, The Netherlands). Deformability is expressed as the elongation index (EI), which is calculated by the height and width of the diffraction pattern that reflects elongation of RBCs within the sample solution during increasing osmolarity (50-600 mOsm/kg) at a constant shear stress of 30Pa. Measurements were carried out according to the manufacturer’s guidelines as described elsewhere.^(4, 5)^ Briefly, 250 µL of whole blood was standardized to a fixed RBC count of 1000•10^6^ and injected into a viscous solution (Iso-elon, RR Mechatronics) and subsequently exposed to an osmolarity gradient and shear of 30Pa. Main outcome parameters are: 1) Omin, which reflects surface area-to-volume ratio; 2) EImax, which is the maximum deformability during changing osmolarity; and 3) Ohyper, which reflects RBC dehydration status; 4) Area under the curve (AUC) (Method figure 1.). For the cell membrane stability test (CMST), whole blood standardized for a fixed RBC count of 200•10^6^ is mixed with 5 mL of the Iso-Elon and sheared at 100Pa during 1 hour. During this test EI is constantly measured and the main outcome parameter ∆EI is derived from the difference in EI between the first 100 seconds and the last 100 seconds of the CMST. The ∆EI is indicative of the ability of the RBC to shed membrane when exposed to shear stress, reflecting membrane health.

**Method figure 1. Ektacytometry parameters.**

**A.** Osmotic gradient ektacytometry showing representative curves of the elongation index for controls (grey) and HS patients (red). **B.** Representative curves for controls (grey) and HS patients (red) of the elongation index during the cell membrane stability test.

*Data sharing statement:*

Further details of analysis and raw data are available upon request.

**References:**

1. Haijes HA, Willemsen M, Van der Ham M, Gerrits J, Pras-Raves ML, Prinsen H, et al. Direct Infusion Based Metabolomics Identifies Metabolic Disease in Patients' Dried Blood Spots and Plasma. Metabolites. 2019;9(1).

2. de Sain-van der Velden MGM, van der Ham M, Gerrits J, Prinsen H, Willemsen M, Pras-Raves ML, et al. Quantification of metabolites in dried blood spots by direct infusion high resolution mass spectrometry. Anal Chim Acta. 2017;979:45-50.

3. van Dooijeweert B, Broeks MH, Verhoeven-Duif NM, van Beers EJ, Nieuwenhuis EES, van Solinge WW, et al. Untargeted metabolic profiling in dried blood spots identifies disease fingerprint for pyruvate kinase deficiency. Haematologica. 2020.

4. Da Costa L, Suner L, Galimand J, Bonnel A, Pascreau T, Couque N, et al. Diagnostic tool for red blood cell membrane disorders: Assessment of a new generation ektacytometer. Blood cells, molecules & diseases. 2016;56(1):9-22.

5. Lazarova E, Gulbis B, Oirschot BV, van Wijk R. Next-generation osmotic gradient ektacytometry for the diagnosis of hereditary spherocytosis: interlaboratory method validation and experience. Clin Chem Lab Med. 2017;55(3):394-402.

**Supplemental Table S2: overview of p-values and isomers**

| **Fig 1/2C. VIP metabolites** | *PLS-DA importance score (1)* | | | | | *PLS-DA importance score (2)* | | *Isomer 1* | | | | *Isomer 2* | | | | | | *Isomer 3* | | | | |  |  | |  |  |  |
| --- | --- | --- | --- | --- | --- | --- | --- | --- | --- | --- | --- | --- | --- | --- | --- | --- | --- | --- | --- | --- | --- | --- | --- | --- | --- | --- | --- | --- |
| Imidazoleacetic acid ribotide | 16,153 | | | | | 15,398 | | * | | | |  | | | | | |  | | | | |  |  | |  |  |  |
| Spermidine | 12,596 | | | | | 11,933 | | * | | | |  | | | | | |  | | | | |  |  | |  |  |  |
| Spermine | 10,273 | | | | | 10,54 | | * | | | |  | | | | | |  | | | | |  |  | |  |  |  |
| Propionylcarnitine | 9,7093 | | | | | 8,9405 | | * | | | |  | | | | | |  | | | | |  |  | |  |  |  |
| 2,3-Diaminopropionic acid | 8,1319 | | | | | 8,6139 | | * | | | |  | | | | | |  | | | | |  |  | |  |  |  |
| Creatine | 7,8802 | | | | | 7,3045 | | Beta-Guanidinopropionic acid | | | |  | | | | | |  | | | | |  |  | |  |  |  |
| N-Acetylgalactosamine | 7,2417 | | | | | 6,7968 | | * | | | |  | | | | | |  | | | | |  |  | |  |  |  |
| 2,3-Diphosphoglyceric acid | 6,6605 | | | | | 6,2612 | | Glyceric acid 1,3-biphosphate | | | |  | | | | | |  | | | | |  |  | |  |  |  |
| N-a-Acetylcitrulline | 6,1522 | | | | | 5,7562 | | Alanyl-Glutamine | | | | Alanyl-Gamma-glutamate | | | | | | Glutaminyl-Alanine | | | | |  |  | |  |  |  |
| Oleoylcarnitine | 5,8766 | | | | | 5,3717 | | Vaccenyl carnitine | | | | Elaidic carnitine | | | | | | 11Z-Octadecenylcarnitine | | | | |  |  | |  |  |  |
| Stearoylcarnitine | 5,1206 | | | | | 4,6816 | | * | | | |  | | | | | |  | | | | |  |  | |  |  |  |
| L-Acetylcarnitine | 4,7738 | | | | | 4,3622 | | * | | | |  | | | | | |  | | | | |  |  | |  |  |  |
| L-Palmitoylcarnitine | 4,756 | | | | | 4,3483 | | * | | | |  | | | | | |  | | | | |  |  | |  |  |  |
| Alanyl-Asparagine | 4,5023 | | | | | 4,6997 | | Asparaginyl-Alanine | | | | Glutaminyl-Glycine | | | | | | Glycyl-Glutamine | | | | |  |  | |  |  |  |
| D-Glyceraldehyde 3-phosphate | | 4,4768 | | | | 4,285 | | Dihydroxyacetone phosphate | | | |  | | | | | |  | | | | |  |  | |  |  |  |
| Linoelaidyl carnitine | 4,2061 | | | | | 3,8438 | | Linoleyl carnitine | | | |  | | | | | |  | | | | |  |  | |  |  |  |
| Lysyl-Valine | 4,0785 | | | | | 3,7283 | | Valyl-Lysine | | | |  | | | | | |  | | | | |  |  | |  |  |  |
| 6-Methylthiopurine 5'-monophosphate ribonucleotide | 3,7393 | | | | | 4,4437 | | * | | | |  | | | | | |  | | | | |  |  |  |  |  |  |
| N1-Acetylspermidine | 3,5074 | | | | | 3,4406 | | N8-Acetylspermidine | | | |  | | | | | |  | | | | |  |  |  |  |  |  |
| Putrescine | 3,5019 | | | | | 3,562 | | 1,4-Butanediammonium | | | |  | | | | | |  | | | | |  |  |  |  |  |  |
| Orotidine | 3,2311 | | | | | 2,9686 | | * | | | |  | | | | | |  | | | | |  |  |  |  |  |  |
| Ureidosuccinic acid | 3,1428 | | | | | 3,0682 | | * | | | |  | | | | | |  | | | | |  |  |  |  |  |  |
| 7-Methylguanine | 3,0294 | | | | | 3,021 | | 3-Methylguanine | | | | 1-Methylguanine | | | | | | N2-Methylguanine | | | | |  |  |  |  |  |  |
| Methionyl-Tyrosine | 2,9513 | | | | | 2,8 | | Tyrosyl-Methionine | | | |  | | | | | |  | | | | |  |  |  |  |  |  |
| Neuraminic acid | 2,9509 | | | | | 2,7679 | | * | | | |  | | | | | |  | | | | |  |  |  |  |  |  |
| Glycylproline | 2,7333 | | | | | 3,0383 | | L-prolyl-L-glycine | | | |  | | | | | | | | |  | | | |  |  |  |  |
| cis-Aconitic acid | 2,7288 | | | | | 2,6058 | | trans-Aconitic acid | | | | Dehydroascorbic acid | | | | | | | | |  | | | |  |  |  |  |
| Glutathione | 2,6537 | | | | | 2,4294 | | * | | | |  | | | | | | | | |  | | | |  |  |  |  |
| Glucosamine-1P | 2,6164 | | | | | 2,4412 | | Glucosamine 6-phosphate | | | | Aminofructose 6-phosphate | | | | | | | | |  | | | |  |  |  |  |
| L-Carnitine | 2,5964 | | | | | 2,3736 | | * | | | |  |  | | | | | | | | | | | |  |  |  |  |
|  |  | | |  |  | | | | |  | | |  | | | | | | | |  |  |  |  |  |  |  |  |
| **Fig 1D. Heatmap** | *T-statistic* | | | *p-value* | | | *FDR* | | *Isomer 1* | | | | | | *Isomer 2* | | | | | *Isomer 3* | | | | *Isomer 4* | | | |  |
| L-Acetylcarnitine | -11,391 | | 1,20E-18 | | | | 2,12E-15 | | * | | | | | |  | | | | |  | | | |  | | | |  |
| Imidazoleacetic acid ribotide | -10,757 | | 2,09E-17 | | | | 1,85E-14 | | * | | | | | |  | | | | |  | | | |  | | | |  |
| cis-Aconitic acid | -10,399 | | 1,06E-16 | | | | 6,27E-14 | | trans-Aconitic acid | | | | | | Dehydroascorbic acid | | | | |  | | | |  | | | |  |
| Propionylcarnitine | -9,3669 | | 1,21E-14 | | | | 5,33E-12 | | * | | | | | |  | | | | |  | | | |  | | | |  |
| Molybdate | -9,1663 | | 3,03E-14 | | | | 1,07E-11 | | * | | | | | |  | | | | |  | | | |  | | | |  |
| Estradiol | 9,1087 | | 3,96E-14 | | | | 1,17E-11 | | 17a-Estradiol | | | | | |  | | | | |  | | | |  | | | |  |
| Oleoylcarnitine | -9,0075 | | 6,31E-14 | | | | 1,46E-11 | | Vaccenyl carnitine | | | | | | Elaidic carnitine | | | | | 11Z-Octadecenylcarnitine | | | |  | | | |  |
| Creatine | -8,9981 | | 6,59E-14 | | | | 1,46E-11 | | Beta-Guanidinopropionic acid | | | | | |  | | | | |  | | | |  | | | |  |
| L-Palmitoylcarnitine | -8,9282 | | 9,09E-14 | | | | 1,79E-11 | | * | | | | | |  | | | | |  | | | |  | | | |  |
| Lysyl-Valine | -8,4544 | | 8,06E-13 | | | | 1,43E-10 | | Valyl-Lysine | | | | | |  | | | | |  | | | |  | | | |  |
| Spermidine | -8,4091 | | 9,93E-13 | | | | 1,48E-10 | | * | | | | | |  | | | | |  | | | |  | | | |  |
| Linoelaidyl carnitine | -8,4068 | | 1,00E-12 | | | | 1,48E-10 | | Linoleyl carnitine | | | | | |  | | | | |  | | | |  | | | |  |
| L-Carnitine | -7,8443 | | 1,33E-11 | | | | 1,81E-09 | | * | | | | | |  | | | | |  | | | |  | | | |  |
| N-Acetylgalactosamine | -7,7965 | | 1,65E-11 | | | | 2,09E-09 | | * | | | | | |  | | | | |  | | | |  | | | |  |
| Imidazole-4-acetaldehyde | -7,4558 | | 7,80E-11 | | | | 9,21E-09 | | * | | | | | |  | | | | |  | | | |  | | | |  |
| N-a-Acetylcitrulline | -7,3869 | | 1,07E-10 | | | | 1,18E-08 | | Alanyl-Glutamine | | | | | | Alanyl-Gamma-glutamate | | | | | Glutaminyl-Alanine | | | | Gamma-glutamyl-Alanine | | | |  |
| Glutamyl-Serine | 7,1869 | | 2,64E-10 | | | | 2,74E-08 | | Serinyl-Glutamate | | | | | |  | | | | |  | | | |  | | | |  |
| Glucosamine-1P | -6,8325 | | 1,29E-09 | | | | 1,27E-07 | | Glucosamine 6-phosphate | | | | | | Aminofructose 6-phosphate | | | | |  | | | |  | | | |  |
| Thymine | -6,7978 | | 1,51E-09 | | | | 1,41E-07 | | Imidazoleacetic acid | | | | | | Imidazol-4-ylacetate | | | | |  | | | |  | | | |  |
| Stearoylcarnitine | -6,7648 | | 1,75E-09 | | | | 1,55E-07 | | * | | | | | |  | | | | |  | | | |  | | | |  |
| N1-Acetylspermidine | -6,6223 | | 3,29E-09 | | | | 2,77E-07 | | N8-Acetylspermidine | | | | | |  | | | | |  | | | |  | | | |  |
| Pyridoxal | 6,2199 | | 1,92E-08 | | | | 1,55E-06 | | Isopyridoxal | | | | | | 3-Methoxyanthranilate | | | | |  | | | |  | | | | |
| 2-Hexaprenyl-3-methyl-5-hydroxy-6-methoxy-1,4-benzoquinol | -6,1225 | | 2,93E-08 | | | | 2,16E-06 | | * | |  | | | | | | | | |  | | | |  | | | | |
| 2,3-Diphosphoglyceric acid | 6,1117 | | 3,07E-08 | | | | 2,16E-06 | | Glyceric acid 1,3-biphosphate | | | | |  | | | | | |  | |  | | | | |  |  |
| Phosphocreatinine | 6,1063 | | 3,14E-08 | | | | 2,16E-06 | | * | |  | | | | | | | | |  | |  | | | | |  |  |
| 4-Pyridoxolactone | 6,1041 | | 3,17E-08 | | | | 2,16E-06 | | Formylanthranilic acid | | | | | | | 5-Pyridoxolactone | | | | Noradrenochrome | |  | | | | |  |  |
| Isobutyryl-L-carnitine | -6,0783 | | 3,55E-08 | | | | 2,32E-06 | | Butyrylcarnitine | |  | | | | | |  | | | | |  | | | | |  |  |
| Threonic acid | -6,0566 | | 3,90E-08 | | | | 2,42E-06 | | * | |  | | | | | |  |  |  |  |  |  |  |  |  |  |  |  |
| Malonic acid | 6,0518 | | 3,98E-08 | | | | 2,42E-06 | | Hydroxypyruvic acid | | | | | | | Tartronate semialdehyde | | |  |  |  |  |  |  |  |  |  |  |
| Orotidine | -6,0446 | | 4,10E-08 | | | | 2,42E-06 | | * | |  | | | | | |  |  |  |  |  |  |  |  |  |  |  |  |
|  |  | |  | |  | | | | |  | | |  | | | | | | | |  |  |  |  |  |  |  |  |
